# A comparison of tiotropium, long-acting β_2_-agonists and leukotriene receptor antagonists on lung function and exacerbations in paediatric patients with asthma

**DOI:** 10.1186/s12931-020-1282-9

**Published:** 2020-01-13

**Authors:** Christian Vogelberg, Stanley Goldstein, LeRoy Graham, Alan Kaplan, Alberto de la Hoz, Eckard Hamelmann

**Affiliations:** 1 Department of Pediatric Pulmonology and Allergy, University Hospital Carl Gustav Carus, Technical University of Dresden, Dresden, Germany; 2 Allergy and Asthma Care of Long Island, Rockville Centre, New York, USA; 3 Pediatric Pulmonology, Children’s Healthcare of Atlanta, Atlanta, GA USA; 4 Family Physician Airways Group of Canada, University of Toronto, Toronto, Ontario Canada; 5 TA Respiratory/Biosimilars Medicine, Boehringer Ingelheim International GmbH, Ingelheim am Rhein, Germany; 6 Klinik für Kinder und Jugendmedizin, Evangelisches Klinikum Bethel, Bielefeld, and Allergy Center of the Ruhr University, Bochum, Germany

**Keywords:** Asthma, Paediatrics, LAMA, LABA, LTRA

## Abstract

Diagnosing and treating asthma in paediatric patients remains challenging, with many children and adolescents remaining uncontrolled despite treatment. Selecting the most appropriate pharmacological treatment to add onto inhaled corticosteroids (ICS) in children and adolescents with asthma who remain symptomatic despite ICS can be difficult. This literature review compares the efficacy and safety of long-acting β_2_-agonists (LABAs), leukotriene receptor antagonists (LTRAs) and long-acting muscarinic antagonists (LAMAs) as add-on treatment to ICS in children and adolescents aged 4–17 years.

A literature search identified a total of 29 studies that met the inclusion criteria, including 21 randomised controlled trials (RCTs) of LABAs versus placebo, two RCTs of LAMAs (tiotropium) versus placebo, and four RCTs of LTRA (montelukast), all as add-on to ICS. In these studies, tiotropium and LABAs provided greater improvements in lung function than LTRAs, when compared with placebo as add-on to ICS. Although exacerbation data were difficult to interpret, tiotropium reduced the risk of exacerbations requiring oral corticosteroids when added to ICS, with or without additional controllers. LABAs and LTRAs had a comparable risk of asthma exacerbations with placebo when added to ICS. When adverse events (AEs) or serious AEs were analysed, LABAs, montelukast and tiotropium had a comparable safety profile with placebo.

In conclusion, this literature review provides an up-to-date overview of the efficacy and safety of LABAs, LTRAs and LAMAs as add-on to ICS in children and adolescents with asthma. Overall, tiotropium and LABAs have similar efficacy, and provide greater improvements in lung function than montelukast as add-on to ICS. All three controller options have comparable safety profiles.

## Lay summary

It can be difficult for doctors to decide which treatment is best to prescribe to children and adolescents with asthma to help reduce their symptoms. In this review, we weigh up the available evidence on three asthma treatments that work in different ways. We looked at two types of inhalers and one type of medicine that is either swallowed as a tablet or granules. The two inhalers helped to improve lung function more than the oral medication, which may be due to their different modes of action. All three treatments were found to be as safe as a placebo.

## Introduction

Asthma is one of the most prevalent chronic diseases in childhood [1], yet diagnosing and treating asthma in children remains challenging. Poor control of asthma in children and adolescents is common and represents a considerable cause of morbidity [2, 3]. In addition to its physical effects, the disease can have an emotional impact on the patient and cause a great burden for patients’ families and the community [1]. There is, therefore, a need for more pharmacological options to improve asthma control in children and adolescents whose symptoms are not fully treated with inhaled corticosteroids (ICS).

**Fig. 1:**
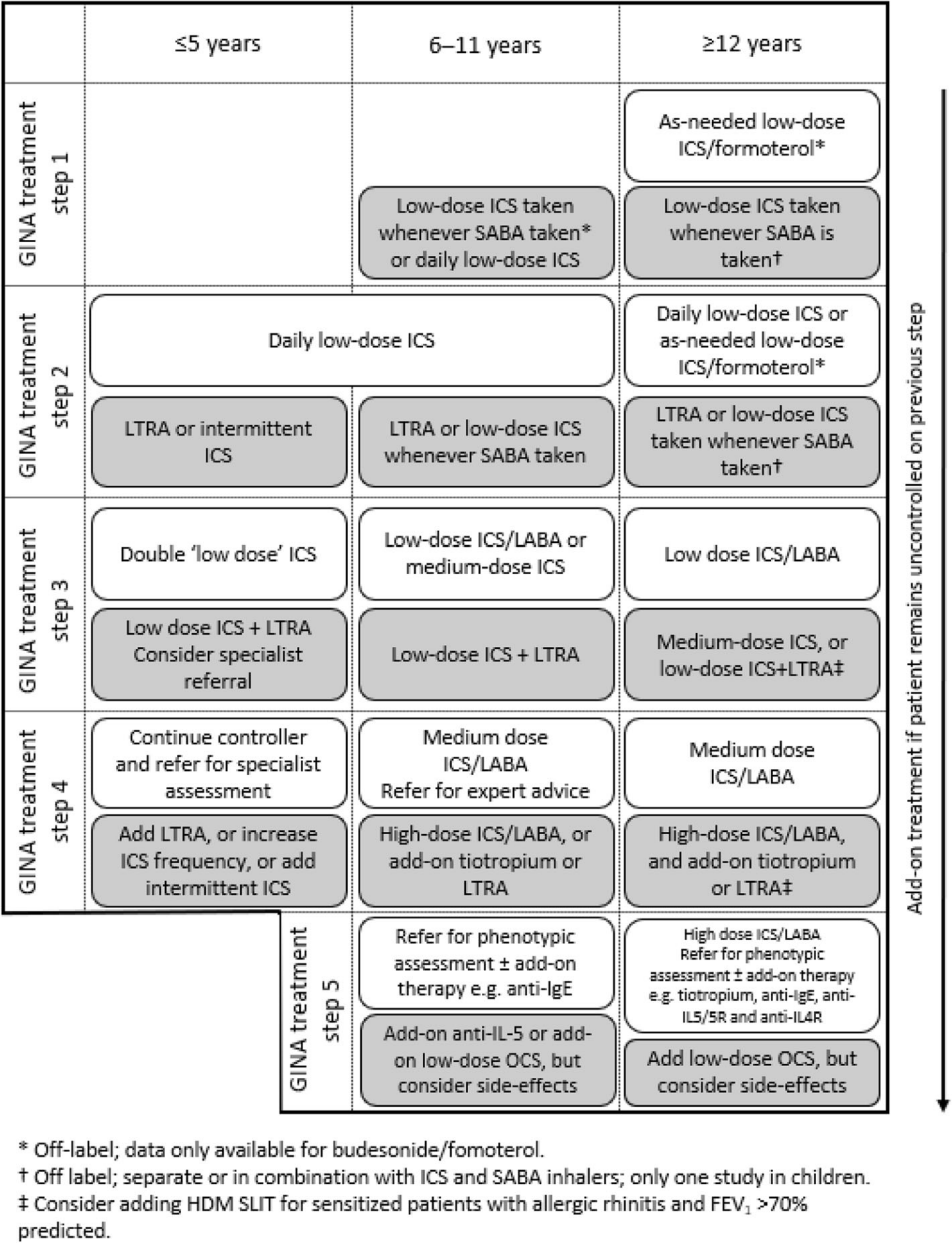
GINA treatment recommendations for patients aged ≥ 5 years, 6–11 years and ≥ 12 years [4]. FEV_1_, forced expiratory volume in 1 s; GINA, Global Initiative for Asthma; HDM, house dust mite; ICS, inhaled corticosteroid; LABA, long-acting β_2_-agonist; LAMA, long-acting muscarinic antagonist; LTRA, leukotriene receptor antagonist; OCS, oral corticosteroids; SABA, short-acting β_2_-agonist; SLIT, sublingual immunotherapy

Selecting the most appropriate add-on treatment to manage and reduce asthma symptoms in children and adolescents whose asthma remains uncontrolled despite treatment can be challenging. The Global Initiative for Asthma (GINA) recommends that patients with asthma who continue to experience symptoms and/or exacerbations on low-dose ICS have their ICS dose increased and combined with long-acting β_2_-agonists (LABAs) or other controllers in a step-wise fashion (Fig. 1). Further controller medications include long-acting muscarinic antagonists (LAMAs; e.g. tiotropium), leukotriene receptor antagonists (LTRAs), theophylline and biologics [4]. GINA also recommends as-needed low-dose ICS/formoterol as reliever therapy in all patients > 12 years of age, with short-acting β_2_-agonists (SABAs) recommended as an alternative reliever medication [4], although it should be noted that the recommendation for children is to ensure additional ICS is taken whenever the SABA reliever is given [4]. The goals of asthma management are aligned across all age groups: namely, to achieve good symptom control, maintain normal activity levels, lung function and development, and minimise future risk of exacerbations and side effects associated with medication [4].

Previous studies have demonstrated the efficacy and safety of LABAs as add-on to ICS compared with placebo [5, 6]. LABAs are available both as single therapy to be taken as add-on to ICS, or as dual therapy, where ICS and LABA are delivered in the same device. Single-therapy LABAs are indicated as add-on treatment to ICS for patients aged from 4 years in Europe and the USA [7–10].

Tiotropium, an alternative add-on treatment to ICS, is a LAMA that is efficacious in clinical trials in adolescents and children with asthma as add-on to ICS [11, 12] or to ICS with other controllers [13, 14]. In the European Union, it is now indicated as add-on maintenance treatment in patients aged 6 years and older with severe asthma who experienced one or more severe asthma exacerbations in the past year [15]. In the USA, tiotropium is indicated in the long-term, once-daily maintenance treatment of asthma in patients aged 6 years and older [16].

The LTRA montelukast is indicated in the treatment of asthma as an add-on therapy in paediatric patients with mild-to-moderate persistent asthma who are inadequately controlled on ICS and in whom SABAs provide inadequate control [17]. It can also be tried as an alternative to ICS in patients with mild-to-persistent asthma who do not have a history of asthma attacks and have trouble using inhaled medications, and is indicated for the prophylaxis of asthma in patients aged at least 2 years [18]. Montelukast oral granules are indicated in patients aged between 6 months and 5 years [19].

Despite the availability of these controller medications, few studies have directly compared their efficacy in adolescents and children with asthma. A number of systematic reviews have compared the effects of LAMAs, LABAs and LTRAs as add-on to ICS in patients with asthma [6, 20–22], although reviews in children aged < 12 years or adolescents aged 12–18 years are limited. Moreover, none have been published that compare the efficacy and safety of all three add-on treatments within one review in patients aged ≤18 years. More systematic reviews and treatment recommendations have been published for patients aged ≥12 years than those for younger patients. As such, there is a need for an up-to-date review of the literature related to the treatments available as add-on to ICS in paediatric patients with asthma.

The aim of this literature review is to compare the efficacy and safety of three controller options (LAMA, LABA and LTRA) as add-on to ICS in adolescents and children aged 4–17 years with asthma. We compare the magnitude of forced expiratory volume in 1 s (FEV_1_) improvements with each drug class, their effects on exacerbations, and the proportion of patients with adverse events (AEs) and serious AEs (SAEs).

## Methods

We carried out an electronic literature search of the Cochrane Database of Systematic Reviews in December 2018 to identify any previously published systematic reviews, which were then manually checked for relevance. We then searched PubMed for articles published since the search date detailed within the systematic review.

The inclusion criteria for this review were randomised controlled trials (RCTs) of at least 4 weeks in duration in children and adolescents aged 4–17 years. The types of intervention included LABA, LAMA or LTRA versus placebo, or versus each other, added onto ICS, compared with the same dose of ICS alone. The primary outcome of interest was lung function, measured using FEV_1_. For FEV_1_, we included percent predicted as well as absolute values, as this has the advantage of removing physical confounding factors, particularly when comparing studies with different age groups of children. Secondary outcomes included exacerbations requiring oral corticosteroids (OCS), and proportion of patients reporting AEs and SAEs.

Data were extracted from published articles in PubMed and publicly available data online. We also checked the reference lists of the systematic reviews for any additional data for endpoints that were not described in the systematic reviews. Results were compared with data from tiotropium trials in paediatric patients (PensieTinA- [NCT01277523], VivaTinA- [NCT01634152], RubaTinA- [NCT01257230] and CanoTinA-asthma® [NCT01634139]).

We used the following search strings:

### Studies of LABA as add-on to ICS

((((((((((clinical trial[MeSH Terms]) OR clinical trial) OR clinical study)))))

**AND** asthma[MeSH Terms]))

**AND** ((((((((Asthma Control Questionnaire) OR ACQ)) OR ((forced expiratory volume) OR FEV)) OR ((exacerbation) OR worsening)) OR adverse event)))))

**AND** ((((((((((((((((((child*) OR paediat*) OR pediat*) OR adolesc*) OR infan*) OR young*) OR preschool*) OR “pre school*”) OR pre-school*))))

**AND** (((((((((seretide) OR symbicort) OR advair) OR viani) OR flutiform))

**OR** (((((((((((glucocorticoids[MeSH Terms]) OR inhaled corticosteroid*) OR budesonide) OR beclomethasone) OR beclometasone) OR fluticasone) OR triamcinolone) OR flunisolide) OR ciclesonide))

**AND** (((((((((adrenergic beta 2 receptor antagonists[MeSH Terms]) OR ((((beta*) AND agonist*)) AND ((long-acting) OR “long acting”))) OR ((((beta*) AND adrenergic*)) AND ((long-acting) OR “long acting”))) OR ((bronchodilat*) AND ((long-acting) OR “long acting”))) OR salmeterol) OR serevent) OR *formoterol) OR foradil) OR vilanterol))))))

**AND** (“2015/02/01”[Date - Publication]: “2018/12/19”[Date - Publication])

### Studies of LTRA as add-on to ICS

((((((((((clinical trial[MeSH Terms]) OR clinical trial) OR clinical study)))))

**AND** asthma[MeSH Terms]))

**AND** ((((((((forced expiratory volume) OR FEV)) OR ((exacerbation) OR worsening)) OR adverse event)))))

**AND** ((((((((((((((((((child*) OR paediat*) OR pediat*) OR adolesc*) OR infan*) OR young*) OR preschool*) OR “pre school*”) OR pre-school*))))

**AND** (((((((((((((glucocorticoids[MeSH Terms]) OR inhaled corticosteroid*) OR budesonide) OR beclomethasone) OR beclometasone) OR fluticasone) OR triamcinolone) OR flunisolide) OR ciclesonide))) AND ((((((((((((leukotriene antagonists[MeSH Terms]) OR LTRA) OR leukotriene*) OR leucotriene*) OR anti-leukotriene*) OR anti-leucotriene*) OR montelukast) OR singulair) OR zafirlukast) OR accolate) OR pranlukast) OR azlaire))))

**AND** (“2014/07/01”[Date - Publication]: “2018/12/19”[Date - Publication])

### Studies of LAMA as add-on to ICS

(((((((((clinical trial[MeSH Terms]) OR clinical trial) OR clinical study)))))

**AND** asthma[MeSH Terms]))

**AND** ((((((((Asthma Control Questionnaire) OR ACQ)) OR ((forced expiratory volume) OR FEV)) OR ((exacerbation) OR worsening)) OR adverse event)))))

**AND** ((((((((((((((((((child*) OR paediat*) OR pediat*) OR adolesc*) OR infan*) OR young*) OR preschool*) OR “pre school*”) OR pre-school*))))

**AND** (((((((((((((glucocorticoids[MeSH Terms]) OR inhaled corticosteroid*) OR budesonide) OR beclomethasone) OR beclometasone) OR fluticasone) OR triamcinolone) OR flunisolide) OR ciclesonide))) **AND** (((((((((((((((((((((((muscarinic) AND antagonist*)))) AND (((long-acting) OR “long acting”)))))) OR ((antagonists, muscarinic[MeSH Terms]) AND (((long-acting) OR “long acting”)))))) OR LAMA) OR glycopyrronium) OR aclidinium) OR tiotropium) OR umeclidinium) OR NVA237) OR seebri) OR LAS34273) OR turdorza) OR pressair) OR eklira) OR genuair) OR spiriva) OR GSK573719)))

The literature searches were reviewed from the title, abstract or descriptors, and all studies that were not RCTs or that clearly did not fit the inclusion criteria were excluded. Data were analysed from the articles deemed appropriate for inclusion. Where appropriate, we performed a meta-analysis using the Cochrane statistical package RevMan 5, assuming equivalence if the risk ratio estimate and its confidence interval (CI) were between 0.9 and 1.1. The risk of bias was assessed using a domain-based evaluation, in line with recommendations provided in the *Cochrane Handbook for Systematic Reviews of Interventions* [23]. Various domains, including allocation concealment and blinding, were judged as being low, unclear or high. Studies were deemed to be of high methodological quality when the reported randomisation and blinding procedures were adequate and at a low risk of bias, with balanced group attrition.

## Results

### Identification of relevant articles

**Fig. 2:**
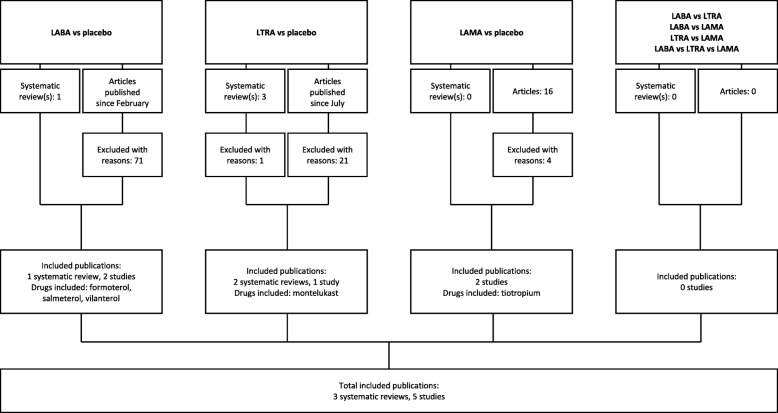
Study selection flow diagram. LABA, long-acting β_2_-agonist; LAMA, long-acting muscarinic antagonist; LTRA, leukotriene receptor antagonist

A literature search identified four systematic reviews (Fig. 2). Of these, one compared RCTs of LABAs as add-on to ICS, published up to February 2015, and was included in the review [24]. Three of the systematic reviews compared LTRAs with placebo as add-on to ICS. Of these, two were included in this review [25, 26], with the most recent studies published up to July 2014. One systematic review comparing LTRAs with placebo [27] was excluded as data from the included studies were already covered in the 2010 systematic reviews. No systematic reviews were identified that compared LAMAs with placebo, or LABAs, LTRAs or LAMAs directly with one another. We reviewed the three systematic reviews and analysed the relevant studies for inclusion in this review.

Additional literature searches identified 73 articles, published since February 2015, comparing LABAs with placebo, of which two met the inclusion criteria for this review [28, 29]. Twenty-three articles published since July 2014 were identified comparing LTRA with placebo, of which one met the inclusion criteria for this review [30]. An additional 16 articles comparing LAMAs with placebo were identified, of which two met the inclusion criteria for this review [11, 31]. We also included two studies in which patients received tiotropium as add-on to ICS plus other controllers, which were not identified in the literature search as the search strings excluded additional controller medications to LAMA [13, 14]. There were no additional studies identified that compared LABAs, LTRAs or LAMAs directly with one another. In total, 29 studies were included in this review.

The designs of all included studies are summarised in Table 1. All studies were randomised, and most were double-blinded and parallel-group in design, ranging from 4 to 54 weeks in duration. Participants were 4–18 years of age. Primary outcomes included safety and lung function.

**Table 1:** Details of the trials included

Study	Reference	Included in previous systematic review	Design	Patient age	Primary outcome
LABA studies					
Formoterol added to budesonide versus budesonide					
SD-039-0719 NCT00646529	Berger 2010 [32]	Yes (Chauhan)	26-week, randomised, open-label, parallel-group, multicentre trial	6–11 years	Safety
SD-039-0725 NCT00646321	Eid 2010 [33]	Yes (Chauhan)	12-week, randomised, double-blind, parallel-group, multicentre trial	6–15 years	PEF
Study 0688	Pohunek 2006 [34]	Yes (Chauhan)	12-week, randomised, double-blind, parallel-group, multicentre trial	4–11 years	Morning PEF
SD-039-0714 ATTAIN	CSR 2003 [35]	Yes (Chauhan)	12-week, randomised, double-blind, parallel-group, multicentre trial	12–17 years	Morning PEF
CHASE 3 NCT02091986	Pearlman 2017 [29]	No	12-week, randomised, double-blind, parallel-group, multicentre trial	6–< 12 years	FEV_1_
	Akpinarli 1999 [36]	Yes (Chauhan)	6-week, randomised, double-blind, parallel-group, multicentre trial	6–14 years	NR
SD-039-0718 NCT00651547		Yes (Chauhan)	12-week, randomised, double-blind, parallel-group, multicentre trial	6–15 years	Morning PEF
SD-039-0682	Morice 2008 [37]	Yes (Chauhan)	12-week, randomised, double-blind, parallel-group, multicentre trial	6–11 years	Morning PEF
Salmeterol added to ICS versus ICS					
SAS30031	Malone 2005 [38]	Yes (Chauhan)	12-week, randomised, double-blind, parallel-group, multicentre trial	4–11 years	Safety
	Carroll 2010 [39]	Yes	8-week, randomised, double-blind, parallel-group, single-centre study	7–18 years	Salbutamol response following cold air challenge
MASCOT	Lenney 2013 [40]	Yes	48-week, randomised, double-blind, parallel-group, multicentre trial	6–14 years	Exacerbations
	Teper 2005 [41]	Yes (Chauhan)	12-month, randomised, double-blind, parallel-group, single-centre trial	6–14 years	NR
SFA100316 NCT00118690	Murray 2011 [42]	Yes (Chauhan)	4-week, randomised, double-blind, parallel-group, multicentre trial	4–17 years	FEV_1_ following exercise
SFA100314	Pearlman 2009 [43]	Yes (Chauhan)	4-week, randomised, double-blind, parallel-group, multicentre trial	4–17 years	FEV_1_ following exercise
	Simons 1997 [44]	Yes (Chauhan)	28-day, randomised, double-blind, crossover, single-centre trial	12–18 years	NR
SAM40012a		Yes (Chauhan)	6-month, randomised, double-blind, parallel-group, multicentre trial	4–11 years	Symptom-free days/nights
SALMP/AH91/D89	Russell 1995 [45]	Yes (Chauhan)	12-week, randomised, double-blind, parallel-group, multicentre trial	4–16 years	Morning PEF % predicted
N/A	Langton Hewer 1995 [46]	Yes (Chauhan)	8-week, randomised, double-blind, parallel-group, single-centre trial	12–17 years	Not identified
	Verberne 1998 [47]	Yes (Chauhan)	54-week, randomised, double-blind, parallel-group, multicentre trial	6–16 years	FEV_1_ and response to methacholine
	Meijer 1995 [48]	Yes (Chauhan)	16-week, randomised, double-blind, parallel-group, single-centre trial	7–15 years	NR
Vilanterol added to fluticasone propionate versus fluticasone propionate					
NCT01573767	Oliver 2016 [28]	No	4-week, randomised, double-blind, parallel-group, multicentre trial	5–11 years	Evening PEF
Tiotropium studies					
Tiotropium added to ICS versus ICS					
RubaTinA-asthma® NCT01257230 2010–021093-11	Hamelmann 2016 [11]	No	48-week, randomised, double-blind, parallel-group, multicentre trial	12–17 years	Peak FEV_1_ response
CanoTinA-asthma® NCT01634139 2011–001758-26	Vogelberg 2018 [31]	No	48-week, randomised, double-blind, parallel-group, multicentre trial	6–11 years	Peak FEV_1_ response
PensieTinA-asthma® NCT01277523 2010–021778-13	Hamelmann 2017 [14]	No	12-week, randomised, double-blind, parallel-group, multicentre trial	12–17 years	Peak FEV_1_ response
VivaTinA-asthma® NCT01634152 2011–001777-43	Szefler 2017 [13]	No	12-week, randomised, double-blind, parallel-group, multicentre trial	6–11 years	Peak FEV_1_ response
Montelukast studies					
	Simons 2001 [49]	Yes (Castro-Rodriguez)	12-week, randomised, double-blind, crossover, multicentre trial	6–14 years	% change in FEV_1_ from baseline
	Miraglia del Giudice 2007 [50]	Yes (Castro-Rodriguez)	1-month, randomised, double-blind, crossover, single-centre study	7–11 years	NR
	Stelmach 2007 [51]	Yes (Zhao)	4-week, randomised, double-blind, parallel-group, single-centre study	6–18 years	4 lung function parameters
NCT01266772	Stelmach 2015 [30]	No	7-month, randomised, double-blind, parallel-group, single-centre study	6–14 years	NR

*FEV*_1_ forced expiratory volume in 1 s, *ICS* inhaled corticosteroid, *NR* not reported, *PEF* peak expiratory flow

An overview of judgements on domains related to risk of bias is reported in Table 2. Most bias items were deemed to be of low or unclear risk.

**Table 2:** Risk of bias summary: review authors’ judgements about each risk of bias item for each included study

	Random sequence generation (selection bias)	Allocation concealment (selection bias)	Blinding (performance bias and detection bias)	Incomplete outcome data (attrition bias)	Selective reporting (reporting bias)	Other bias
LABA added to ICS versus ICS						
Akpinarli 1999	?	?	+	?	+	?
Berger 2010	+	+	–	+	?	?
Carroll 2010	?	+	+	+	+	+
Eid 2010a^a^	?	?	+	?	–	+
Eid 2010b^a^	?	?	+	?	–	+
Langton Hewer 1995	?	?	+	?	+	?
Lenney 2013	+	+	+	+	+	+
Malone 2005	+	+	+	–	+	+
Meijer 1995	?	?	+	?	?	?
Morice 2008a^a^	+	?	+	?	–	+
Morice 2008b^a^	+	?	+	?	–	+
Murray 2011	?	?	+	+	+	+
Oliver 2016	+	?	+	+	?	+
Pearlman 2009	+	?	+	+	+	+
Pohunek 2006a^a^	+	+	+	?	?	+
Pohunek 2006b^a^	+	+	+	?	?	+
Russell 1995	+	+	+	–	+	?
SAM40012	+	+	+	?	?	?
SD 0390714	?	?	+	?	?	+
SD 0390718	?	?	+	?	?	+
Simons 1997	+	?	+	+	+	?
Teper 2005	?	?	+	?	?	?
Verberne 1998a^a^	+	+	+	?	+	?
Verberne 1998b^a^	+	+	+	?	+	?
Tio added to ICS versus ICS						
Hamelmann 2016	+	+	+	+	+	+
Vogelberg 2018	+	+	+	+	+	+
Tio added to ICS with other controllers versus ICS with other controllers						
Hamelmann 2017	+	+	+	+	+	+
Szefler 2017	+	+	+	+	+	+
LTRA added to ICS versus ICS						
Simons 2001	?	+	+	+	?	+
Miraglia del Giudice 2007	+	+	+	+	?	+
Stelmach 2007	+	?	+	+	?	+
Stelmach 2015	+	+	+	+	?	+

Key: + low risk of bias; − high risk of bias;? unclear risk of bias

*ICS* inhaled corticosteroid, *LABA* long-acting β_2_-agonist, *LTRA* leukotriene receptor antagonist, *Tio* tiotropium

^a^‘a’ and ‘b’ refer to different treatment arms of the same study

### FEV_1_ results

The LABA studies included in the Cochrane meta-analysis present a combination of peak and trough FEV_1_ measurements, and some articles do not specify at what time point the measurement was taken [24]. For this reason, we present both peak and trough FEV_1_ response data where available.

### FEV_1_: absolute difference in litres

**Fig. 3:**
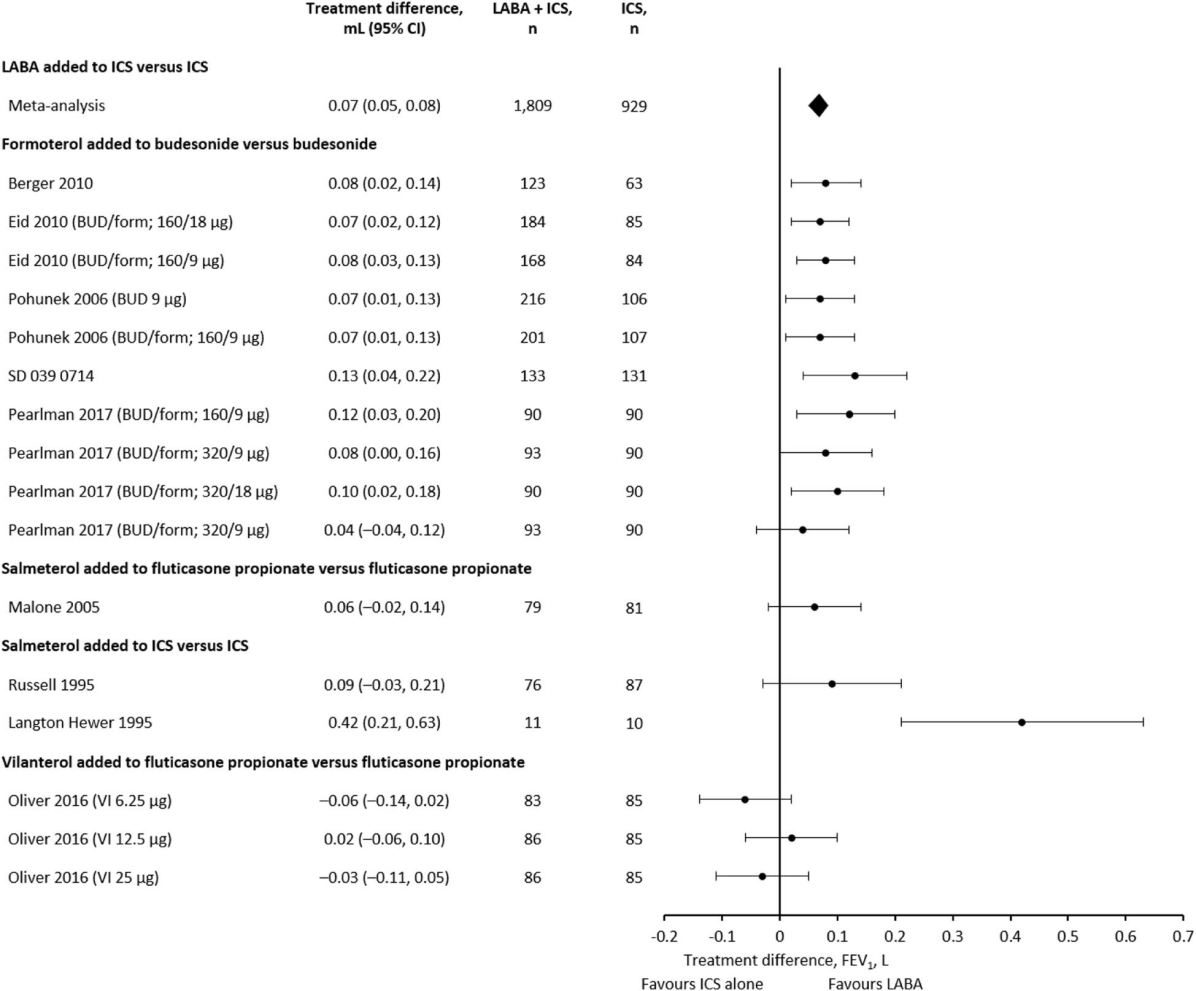
Treatment difference in FEV_1_ response between LABA added to ICS and ICS alone. BUD, budesonide; CI, confidence interval; FEV_1_, forced expiratory volume in 1 s; form, formoterol; ICS, inhaled corticosteroid; LABA, long-acting β_2_-agonist; VI, vilanterol

We performed a meta-analysis of nine LABA studies. There was a treatment difference in FEV_1_ of 0.07 L (95% CI 0.05, 0.08) (Fig. 3). Excluding the two outliers (a vilanterol study that found no improvement [− 0.06 to 0.02 L] [28] and a very small [*n* = 21] salmeterol study [0.42 L (95% CI 0.21, 0.63)] [46]), mean treatment differences were 0.04–0.13 L (Fig. 3). None of the included LTRA studies presented data for change from baseline in litres.

**Fig. 4:**
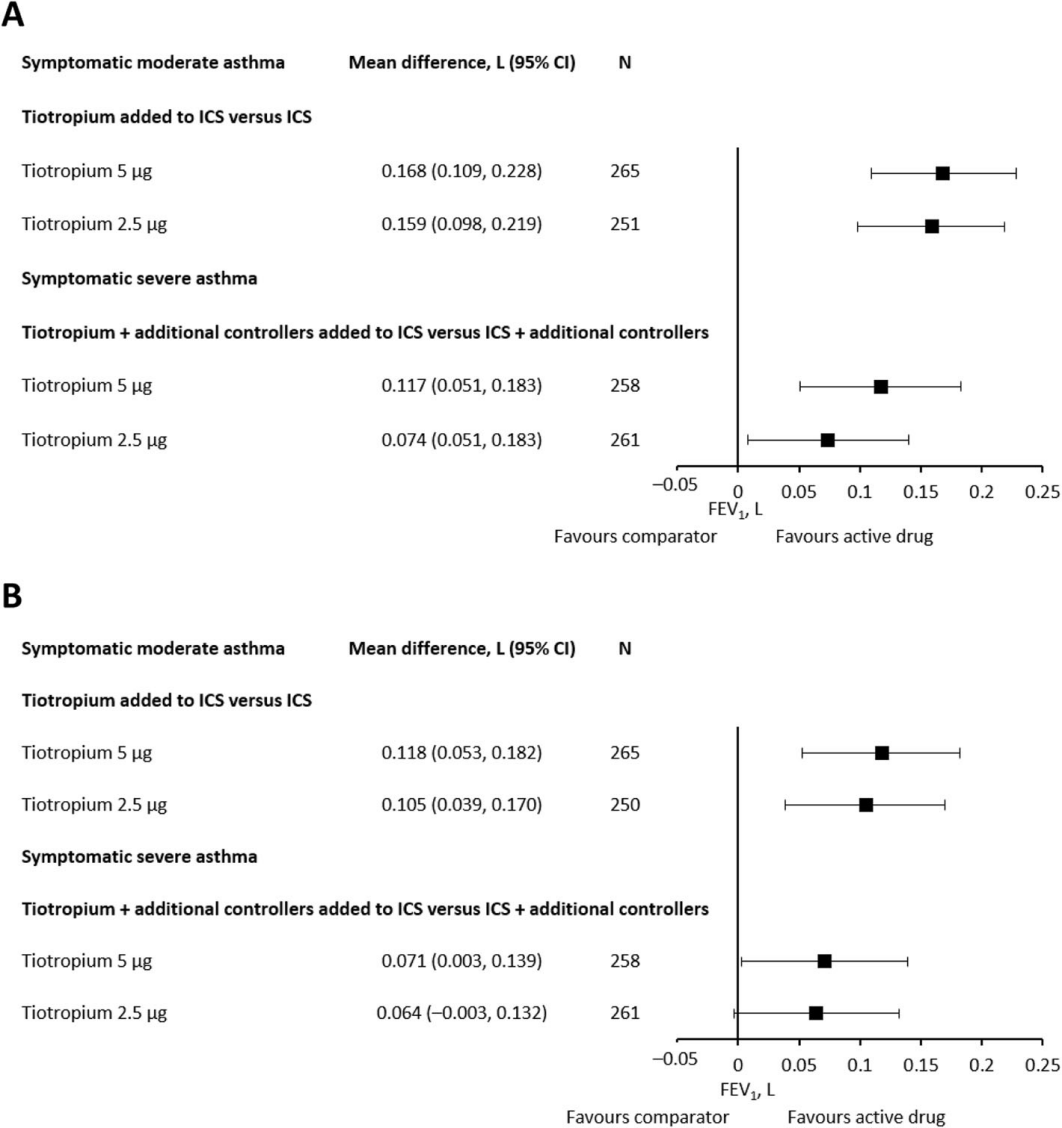
Pooled treatment difference in peak (**a**) and trough (**b**) FEV_1_ response between tiotropium Respimat® and placebo added to ICS for patients with symptomatic moderate asthma and patients with symptomatic severe asthma. CI, confidence interval; FEV_1_, forced expiratory volume in 1 s; ICS, inhaled corticosteroid

For the LAMA studies, we pooled the data for studies where tiotropium was the only add-on therapy (no additional LABA add-on therapy permitted) (RubaTinA-asthma® and CanoTinA-asthma®) [11, 31] and presented both peak and trough results for tiotropium Respimat® 5 μg and 2.5 μg (Fig. 4). Peak FEV_1_ was defined as the maximum FEV_1_ within 3 h after dosing and trough FEV_1_ was defined as the pre-dose FEV_1_ measured 24 h after the previous drug administration and 10 min prior to the evening dose of the patient’s usual asthma medication. We did the same for studies where tiotropium Respimat® was the third or even fourth controller (PensieTinA-asthma® and VivaTinA-asthma®) (Fig. 4). None of the included studies investigated tiotropium delivered via the HandiHaler® device [13, 14].

FEV_1_ improvements versus placebo with tiotropium Respimat® as add-on to ICS in studies of children and adolescents with symptomatic moderate asthma were 0.159–0.168 L for peak FEV_1_ and 0.105–0.118 L for trough FEV_1_ (Fig. 4). For studies in children and adolescents with symptomatic severe asthma, FEV_1_ improvements versus placebo were 0.074–0.117 L for peak FEV_1_ and 0.064–0.071 L for trough FEV_1_ (Fig. 4).

### FEV_1_ response: percent predicted

The Cochrane analysis of LABA studies (Table 3) found an improvement in FEV_1_ percent predicted with LABAs added to ICS versus ICS of 2.99% (95% CI 0.86, 5.11; *n* = 534) [24]. Results from individual LABA studies are also detailed in Table 3. Improvements in peak FEV_1_ percent predicted with tiotropium added to ICS versus ICS were 4.07–7.70%, and 2.85–5.05% for trough FEV_1_; improvements with tiotropium added to ICS with other controllers were 1.64–6.33% for peak FEV_1_ and 0.83–3.85% for trough FEV_1_.

**Table 3:** Mean difference in FEV_1_% predicted

Drug	Age, years	n^a^	Mean difference FEV_1_, % predicted (95% CI) active drug vs placebo
LABA added to ICS versus ICS, FEV_1_ response (Cochrane analysis: Chauhan 2015)		534	2.99 (0.86, 5.11)^b^
Formoterol added to ICS versus ICS			
Akpinarli 1999 Formoterol 12 μg BID add-on to ICS 400–800 μg/day	6–14	32	2.00 (−24.10, 28.10)^b^
Salmeterol added to beclomethasone dipropionate versus beclomethasone dipropionate			
Verberne 1998 Salmeterol/beclomethasone dipropionate 50/200 μg BID vs beclomethasone dipropionate 200 μg BID	6–16	117	3.08 (−0.49, 6.65)^b^
Meijer 1995 Salmeterol 50 μg BID + beclomethasone dipropionate 250 μg BID	7–15	39	3.60 (−2.94, 10.14)^b^
Salmeterol added to fluticasone propionate versus fluticasone propionate			
Carroll 2010 Fluticasone/salmeterol 100/50 BID vs fluticasone 100 μg BID	7–18	37	5.20 (−1.04, 11.44)^b^
Lenney 2013 Fluticasone propionate/salmeterol 100/50 μg BID vs fluticasone propionate 100 μg BID	6–14	21	15.42 (1.51, 29.33)^b^
Teper 2005 Fluticasone/salmeterol 125/25 μg BID vs fluticasone 125 μg BID	6–14	82	−0.40 (−5.03, 4.23)^b^
Salmeterol added to ICS versus ICS			
Russell 1995 Salmeterol 50 μg BID add-on to ICS 400–2400 μg/day	4–16	206	3.40 (−1.54, 8.34)^b^
Tiotropium in moderate asthma			
Tiotropium 5 μg Add-on to 400–800 μg/day budesonide (200–800 μg/day for patients aged 12–14 years)	12–17	268 268	Trough: 3.205 (0.209, 6.201) Peak: 4.492 (1.700, 7.285)
Tiotropium 2.5 μg Add-on to 400–800 μg/day budesonide (200–800 μg/day for patients aged 12–14 years)	12–17	256 257	Trough: 2.850 (−0.229, 5.929) Peak: 4.066 (1.208, 6.924)
Tiotropium 5 μg Add-on to 200–400 μg budesonide	6–11	260 260	Trough: 4.439 (1.207, 7.671) Peak: 6.521 (3.717, 9.325)
Tiotropium 2.5 μg Add-on to 200–400 μg budesonide	6–11	257 257	Trough: 5.048 (1.811, 8.285) Peak: 7.698 (4.892, 10.505)
Tiotropium in severe asthma			
Tiotropium 5 μg Add-on to high-dose ICS^c^ + ≥1 controller or medium-dose ICS^d^ + ≥2 controllers	12–17	262 262	Trough: 0.827 (−2.354, 4.008) Peak: 1.643 (−1.252, 4.539)
Tiotropium 2.5 μg Add-on to high-dose ICS^c^ + ≥1 controller or medium-dose ICS^d^ + ≥2 controllers	12–17	258 258	Trough: 3.283 (0.075, 6.491) Peak: 3.106 (0.188, 6.024)
Tiotropium 5 μg Add-on to > 400 μg budesonide + ≥1 controller or 200–400 μg budesonide + ≥2 controllers	6–11	258 258	Trough: 3.848 (0.576, 7.120) Peak: 6.325 (3.264, 9.385)
Tiotropium 2.5 μg Add-on to > 400 μg budesonide + ≥1 controller or 200–400 μg budesonide + ≥2 controllers	6–11	265 265	Trough: 2.350 (−0.909, 5.609) Peak: 3.587 (0.540, 6.634)
Montelukast			
Castro-Rodriguez 2010 Meta-analysis: Montelukast 5 mg QD Add-on to 200–800 μg/day budesonide	5–18	188^a^	0.09 (−0.07, 0.25)^b^
Simons 2001 Montelukast 5 mg QD + budesonide 200 μg BID vs budesonide 200 μg BID	6–14	279	1.3 (− 0.1, 2.7)^b^
Miraglia del Giudice 2007 Montelukast 5 μg QD + budesonide 200 μg BID vs budesonide 200 μg BID	7–11	48	10.8 (NR)^b^
Zhao 2015 Network meta-analysis: Montelukast 4–10 mg QD add-on to 100–200 μg/day budesonide	≤18	NR	
Stelmach 2007 Montelukast 5–10 μg QD + 200 μg budesonide BID vs 200 μg budesonide BID	6–18	76	2.6 (NR)^b^
Stelmach 2015 Montelukast 5 mg QD add-on to 200–600 μg budesonide^e^	6–14	76	2.5 (NR)^b,f^

*BID* twice daily, *CI* confidence interval, *FEV*_1_ forced expiratory volume in 1 s, *ICS* inhaled corticosteroid, *LABA* long-acting β_2_-agonist, *NR* not reported, *QD* once daily

^a^Total n number for the treatment arms being compared. ^b^Time of measurement relevant to dosing (peak/trough) not specified. ^c^High-dose ICS defined as > 400 μg budesonide (aged 12–14 years)/800–1600 μg budesonide (aged 15–17 years). ^d^Medium-dose ICS defined as 200–400 μg budesonide (aged 12–14 years)/400–800 μg budesonide (aged 15–17 years). ^e^ICS dose was adjusted during the course of this study. ^f^Change from placebo was not significantly different (*P* = 0.229)

The treatment difference with montelukast added to ICS compared with ICS alone varied, with the systematic review finding an improvement of 0.09% (95% CI − 0.07 to 0.25; *n* = 188) [25] and individual studies mostly ranging from 1.3 to 2.6%. One single-centre study found an improvement of 10.8% with montelukast compared with ICS, but this was a small, 4-week study (*n* = 24), and no confidence intervals or statistical comparison was available [50].

### Exacerbations requiring OCS

The Cochrane analysis of LABA studies (*n* = 1669) found no difference in the risk of exacerbations requiring OCS between LABAs plus ICS compared with ICS alone (risk ratio 0.95; 95% CI 0.70, 1.28) (Table 4) [24]. The individual studies were quite variable, with study durations of 4–54 weeks. We found no additional studies reporting on exacerbations requiring OCS in our literature search.

**Table 4:** Exacerbations requiring oral corticosteroids

Drug	Time period	n^a^	Number of patients with exacerbations requiring OCS, n/N (%)		Exacerbations requiring OCS^b^
			Active treatment	Comparator	Risk ratio (95% CI)
Cochrane analysis of LABA studies (Chauhan 2015)		1669			0.95 (0.70, 1.28)
Formoterol added to ICS versus ICS					
Eid 2010 Budesonide/formoterol 160/18 μg daily vs budesonide 160 μg QD	12 weeks	267	15/183 (8.2)	13/84 (15.5)	0.53 (0.26, 1.06)
Eid 2010 Budesonide/formoterol 160/9 μg daily vs budesonide 160 μg daily	12 weeks	252	33/168 (19.6)	13/84 (15.5)	1.27 (0.71, 2.28)
Salmeterol added to ICS versus ICS					
Langton Hewer 1995 Salmeterol 100 μg BID add-on to usual ICS (baseline mean 400 μg)	8 weeks	23	3/11 (27.2)	3/12 (25.0)	1.09 (0.28, 4.32)
Lenney 2013 Fluticasone propionate/salmeterol 100/50 μg BID vs fluticasone propionate 100 μg BID	48 weeks	26	5/15 (33.3)	1/11 (9.1)	3.67 (0.50, 27.12)
Malone 2005 Salmeterol/fluticasone 50/100 μg BID vs fluticasone 100 μg BID	3 months	203	2/101 (2.0)	3/102 (2.9)	0.67 (0.11, 3.94)
Murray 2011 Salmeterol/fluticasone 50/100 μg BID vs fluticasone 100 μg BID	4 weeks	231	2/113 (1.8)	1/118 (0.8)	2.09 (0.19, 22.71)
Pearlman 2009 Salmeterol/fluticasone 50/100 μg BID vs fluticasone 100 μg BID	4 weeks	248	1/124 (0.8)	1/124 (0.8)	1.00 (0.06, 15.81)
Simons 1997 Salmeterol 50 μg QD add-on to BDP 200–400 μg/day	4 weeks	32	0/16 (0.0)	1/16 (6.3)	0.33 (0.01, 7.62)
Verberne 1998 Salmeterol/BDP 50/200 μg BID vs BDP 200 μg BID	54 weeks	117	10/60 (16.7)	10/57 (17.5)	0.95 (0.43, 2.11)
Russell 1995 Salmeterol 50 μg BID add-on to ICS 400–2400 μg/day	12 weeks	198	16/99 (16.2)	18/99 (18.2)	0.89 (0.48, 1.64)
Tiotropium added to ICS versus ICS					Hazard ratio (95% CI)
Hamelmann 2016 Tiotropium 5 μg add-on to 400–800 μg/day budesonide (200–800 μg/day for patients aged 12–14 years)	48 weeks	272	2/134 (1.5)	9/138 (6.5)	0.23 (0.05, 1.08)^c^
Hamelmann 2016 Tiotropium 2.5 μg add-on to 400–800 μg/day budesonide (200–800 μg/day for patients aged 12–14 years)	48 weeks	263	5/125 (4.0)	9/138 (6.5)	0.63 (0.21, 1.87)^c^
Vogelberg 2018 Tiotropium 5 μg add-on to 200–400 μg budesonide	48 weeks	266	7/135 (5.2)	6/131 (4.6)	1.14 (0.38, 3.39)^c^
Vogelberg 2018 Tiotropium 2.5 μg add-on to 200–400 μg budesonide	48 weeks	266	7/135 (5.2)	6/131 (4.6)	1.14 (0.38, 3.38)^c^
Tiotropium added to ICS plus other controller(s) versus ICS plus other controller(s)					
Hamelmann 2017 Tiotropium 5 μg add-on to high-dose ICS^d^ + ≥1 controller or medium-dose ICS^e^ + ≥2 controllers	12 weeks	265	2/130 (1.5)	1/135 (0.7)	2.06 (0.19, 22.70)^c^
Hamelmann 2017 Tiotropium 2.5 μg add-on to high-dose ICS^d^ + ≥1 controller or medium-dose ICS^e^ + ≥2 controllers	12 weeks	262	1/127 (0.8)	1/135 (0.7)	1.06 (0.07, 16.95)^c^
Szefler 2017 Tiotropium 5 μg add-on to > 400 μg budesonide + ≥1 controller or 200–400 μg budesonide + ≥2 controllers	12 weeks	264	7/130 (5.4)	8/134 (6.0)	1.01 (0.35, 2.88)^c^
Szefler 2017 Tiotropium 2.5 μg add-on to > 400 μg budesonide + ≥1 controller or 200–400 μg budesonide + ≥2 controllers	12 weeks	270	3/136 (2.2)	8/134 (6.0)	0.40 (0.10, 1.55)^c^
Montelukast added to ICS versus ICS					
Castro-Rodriguez 2010 systematic review Montelukast 5 mg add-on to 200–800 μg/day budesonide	NR	NR	NR	NR	Risk ratio (95% CI) 0.53 (0.10, 2.74)^f^
Zhao 2015 network meta-analysis Montelukast 4–10 mg add-on to 100–200 μg/day budesonide	4–16 weeks	NR	NR	NR	Odds ratio (95% CI) 0.94 (0.58, 1.45)
Stelmach 2015 Montelukast 5 mg add-on to 200–600 μg budesonide^g^	7 months	76	NR	NR	Odds ratio (95% CI) 0.26 (0.09, 0.76)

*BDP* beclomethasone dipropionate, *BID* twice daily, *CI* confidence interval, *ICS* inhaled corticosteroid, *LABA* long-acting β_2_-agonist, *NR* not recorded, *OCS* oral corticosteroid, *QD* once daily

^a^Total n number for the treatment arms being compared. ^b^Risk ratio or odds ratio as noted. ^c^Data on file. ^d^ > 400 μg budesonide (aged 12–14 years)/800–1600 μg budesonide (aged 12–17 years). ^e^200–400 μg budesonide (aged 12–14 years)/400–800 μg budesonide (aged 15–17 years). ^f^Authors note evidence of statistical heterogeneity for this analysis. ^g^ICS dose was adjusted during the course of this study

Risk ratios were not available for the tiotropium studies, but the proportion of patients with exacerbations requiring OCS was low in all of the studies (Table 4). Tiotropium provided improvements in time to first exacerbation requiring OCS when added onto ICS versus placebo, with hazard ratios of 0.23–1.14, and 0.40–2.06 when added on to other controllers.

The systematic review of the LTRA studies showed no difference between montelukast and placebo on top of ICS, but the authors noted that there was evidence of statistical heterogeneity [25]. The network meta-analysis found no difference between montelukast and placebo (odds ratio 0.94; 95% CI 0.58, 1.45) [26]. One 7-month study found fewer exacerbations with montelukast than with placebo as add-on to ICS (odds ratio 0.26; 95% CI 0.09, 0.76) [30].

### Adverse events and serious adverse events

The proportion of patients experiencing AEs or SAEs with the addition of LABA to ICS was broadly similar, with some variations in the proportion of patients with AEs or SAEs between studies (Table 5).

**Table 5:** AEs and SAEs

Drug	Duration	n^a^	Number of patients with AE, n (%)		Number of patients with SAE, n (%)	
			Active	Comparator	Active	Comparator
LABAs added to ICS versus ICS						
Berger 2010 Budesonide/formoterol pMDI 320/9 μg BID	26 weeks	186	104 (84.6)	54 (85.7)	2 (1.6)	1 (1.6)
Eid 2010 Budesonide/formoterol 160/18 μg daily	12 weeks	184	120 (65.2)	100 (59.2)	2 (1.1)	1 (0.6)
Eid 2010 Budesonide/formoterol 160/9 μg daily	12 weeks	168	104 (61.9)	100 (59.2)	3 (1.8)	1 (0.6)
Langton Hewer 1995 Salmeterol 100 μg BID	8 weeks	24	10 (91)	9 (75)	NR	NR
Malone 2005 Salmeterol/fluticasone 50/100 μg BID	3 months	203	101 (59)	102 (57)	NR	NR
Morice 2008a Budesonide/formoterol 160/9 μg DPI BID	12 weeks	419	100 (47)	81 (39)	2 (0.9)	0
Morice 2008b Budesonide/formoterol 160/9 μg MDI BID	12 weeks	410	92 (45)	81 (39)	3 (1.5)	0
Murray 2011 Salmeterol/fluticasone 50/100 μg BID	4 weeks	231	20 (18)	25 (21)	0	0
Pearlman 2009 Salmeterol/fluticasone 50/100 μg BID	4 weeks	248	37 (30)	35 (28)	0	0
SD 0390718 Formoterol/budesonide 9/80 μg BID	12 weeks	273	90 (70.3)	92 (63.4)	0	0
Verberne 1998a Salmeterol/beclomethasone dipropionate 50/200 μg BID	54 weeks	117	59 (98)	52 (93)	NR	NR
Russell 1995 Salmeterol 50 μg BID	12 weeks	206	74 (75)	81 (76)	10 (10)	13 (12)
SD 0390714 Formoterol/budesonide 4.5/160 μg BID	12 weeks	270	66 (49)	65 (49)	1 (0.7)	1 (0.7)
SAM40012 Salmeterol/fluticasone propionate 50/100 μg BID	6 months	362	99 (55)	111 (61)	2 (1)	1 (< 1)
Pearlman 2017	12 weeks					
Budesonide/formoterol 160/9 μg BID		18	42 (46.7)	40 (44.4)	0	2 (2.2)
Budesonide/formoterol 160/4.5 μg BID		183	41 (44.1)	40 (44.4)	0	2 (2.2)
Oliver 2016	4 weeks					
Vilanterol 6.25 μg QD		229	33 (29)	25 (22)	NR	NR
Vilanterol 12.5 μg QD		228	37 (33)	25 (22)		
Vilanterol 25 μg QD		229	32 (28)	25 (22)		
Tiotropium added to ICS vs ICS						
Hamelmann 2016	48 weeks					
Tiotropium 5 μg QD		272	84 (62.7)	82 (59.4)	3 (2.2)	2 (1.4)
Tiotropium 2.5 μg QD		263	79 (63.2)	82 (59.4)	2 (1.6)	2 (1.4)
Vogelberg 2018	48 weeks					
Tiotropium 5 μg QD		266	82 (60.7)	89 (67.9)	1 (0.7)	6 (4.6)
Tiotropium 2.5 μg QD		266	86 (63.7)	89 (67.9)	3 (2.2)	6 (4.6)
Tiotropium added to ICS with other controllers vs ICS with other controllers						
Hamelmann 2017	12 weeks					
Tiotropium 5 μg QD		265	43 (33.1)	48 (35.6)	2 (1.5)	0
Tiotropium 2.5 μg QD		262	42 (33.1)	48 (35.6)	1 (0.8)	0
Szefler 2017	12 weeks					
Tiotropium 5 μg QD		264	56 (43.1)	66 (49.3)	4 (3.1)	2 (1.5)
Tiotropium 2.5 μg QD		270	59 (43.4)	66 (49.3)	2 (1.5)	2 (1.5)
LTRAs added to ICS vs ICS						
Simons 2001 Montelukast 5 mg	4 weeks (crossover trial)	279	277 (42)	270 (45)	NR	NR

*AE* adverse event, *BID* twice daily, *DPI* dry powder inhaler, *ICS* inhaled corticosteroid, *LABA* long-acting β_2_-agonist, *MDI* metered-dose inhaler, *pMDI* pressurised metered-dose inhaler, *QD* once daily, *SAE* serious adverse event

^a^Total n number for the treatment arms being compared

There was no increase in the number of patients with AEs or SAEs with tiotropium compared with placebo as add-on to ICS or add-on to ICS plus other controllers (Table 5).

There were limited data on the number of patients with AEs in the montelukast analyses; the study that did report the proportion of patients with AEs showed no significant difference between montelukast and placebo as add-on to ICS (Table 5). There were insufficient data to make a comment on SAEs in the montelukast trials.

### Efficacy and safety of tiotropium Respimat® as add-on to ICS and additional controller medications

In studies where tiotropium Respimat® was added onto ICS and additional controller medications (PensieTinA-asthma® and VivaTinA-asthma®) [13, 14], the effect size for both lung function and exacerbations requiring OCS was comparable with the studies where tiotropium was the only controller [11, 31], or where LABA or LTRA were added onto ICS [24–26, 28–30]. In addition, the studies demonstrated comparable safety with placebo [13, 14].

## Discussion

In this literature review, the addition of once-daily tiotropium (with or without other controllers) and twice-daily LABAs to ICS in children and adolescents provided similar improvements in lung function [11, 13, 14, 24, 28, 29, 31], and greater improvements than with once-daily LABA vilanterol added onto ICS [28]. Data reporting on the effect of LTRAs as add-on to ICS on lung function were somewhat inconsistent, yet a previous systematic review found no improvement with montelukast compared with placebo when added to ICS [25], so it may be appropriate to suggest that twice-daily LABAs and tiotropium are more effective at improving lung function in adolescents and children as add-on to ICS. This assumption could be further clarified if future studies directly compared tiotropium, LABAs and LTRAs as add-on to ICS.

An additional endpoint that we analysed in this review was asthma exacerbations. However, the exacerbation data were more difficult to interpret, as the studies were of different durations and not necessarily powered to show a treatment difference in exacerbation frequency. Powering a study in paediatric patients to assess asthma exacerbations may present ethical considerations, with patients receiving placebo or care that is inconsistent with the best proven method, potentially being exposed to unnecessary risk and harm, especially where exacerbation events are expected [52]. In addition, not all studies included a risk ratio, making the comparison of data difficult. However, in the tiotropium trials, where exacerbations were included as a safety endpoint, it was possible to demonstrate that tiotropium provided a reduction in the risk of exacerbations requiring OCS when added onto ICS, either alone or with additional controller treatments, compared with placebo [11, 13, 14, 31]. Although the results from the individual studies of LABA as add-on to ICS varied, the previously published Cochrane review by Chauhan et al. suggested that LABAs and placebo have a comparable risk of asthma exacerbation [24]. In regards to the effect of LTRAs on asthma exacerbations, the data were more inconclusive. The one RCT included on LTRAs reported that montelukast reduced the risk of exacerbations compared with placebo. However, the sample size was small, with only 76 participants [30]. The two systematic reviews reported no reduction in the risk of exacerbations compared with placebo; however, the width of the CIs suggests a large spread of data [25, 26]. It could therefore be suggested that the highest quality of evidence was for the trials investigating LABA or LAMA as add-on to ICS.

The safety data showed no increase in the proportion of patients reporting AEs or SAEs with LABAs or with tiotropium when added to ICS [11, 13, 14, 24, 28, 29, 31]. The available data for LTRAs were limited, but suggested no increase in the proportion of patients with AEs with montelukast compared with placebo as add-on to ICS [49]. However, it should be noted that previous post-marketing studies have suggested that paediatric patients receiving montelukast are more likely to report neuropsychiatric AEs than those receiving ICS [53, 54]. Therefore, the results from this review indicate that LABAs, LTRAs and LAMAs all have a comparable safety profile to placebo, but other real-world and post-marketing evidence should also be considered.

This literature review aims to provide an up-to-date overview of the efficacy and safety of three classes of drugs that are options for adding onto ICS in adolescents and children with asthma. The strength of the study is that this is the first literature review and meta-analysis to collate and compare the efficacy and safety of LABAs, LTRAs and LAMAs in children and adolescents in one review. Previous reviews have compared the efficacy and safety of LABAs and LAMAs, or LABAs and LTRAs, in adolescents aged over 12 years and in adults, but none has compared all three therapeutic options in one review, and none has done so for this patient population in children and adolescents aged 4–17 years.

We have focused on a limited number of endpoints that are considered important in the treatment of asthma such as lung function, exacerbations and AEs. However, there is considerable variability in the methodology and definition of these endpoints between studies, making the comparison of data more difficult. There were only a limited number of montelukast studies in children that met the inclusion criteria, so LTRA data are lacking for some endpoints. For example, for the LABA studies, we were able to perform a meta-analysis of absolute change in lung function in litres, but LTRA studies only reported lung function change in percent predicted. Moreover, when extracting the FEV_1_ data from the various studies, the time point of the measurement in relation to drug administration (i.e. peak/trough) was not always clear. Only the LAMA studies reported whether FEV_1_ was peak (defined as the maximum FEV_1_ within 3 h after dosing) or trough FEV_1_ (defined as the pre-dose FEV_1_ measured 24 h after the previous drug administration and 10 min prior to the evening dose of the patient’s usual asthma medication). As Fig. 4 demonstrates, there are differences between the responses depending on when the measurement is taken, with peak FEV_1_ (Fig. 4a) values higher than the equivalent trough FEV_1_ (Fig. 4b) values. Therefore, it is possible that some of the between-study differences in FEV_1_ response for LABAs and LTRAs may be attributable to the time point at which the measurement was taken, but this cannot be confirmed.

In light of the extension of the tiotropium label and the most recent treatment guidelines for children with asthma [4], the results provide support for the use of tiotropium as add-on therapy in adolescents and children with asthma aged 4–17 years. The results are in agreement with those of a recently published systematic review that compared LABAs with LAMAs in patients aged over 12 years [22]. The authors reported that use of LAMA as add-on to ICS was associated with a lower risk of asthma exacerbations compared with placebo, and had a comparable benefit to LABA on lung function. The authors note that their review was designed and conducted in patients aged 12 years and over because tiotropium was not approved in patients aged less than 12 years at the time the study was undertaken [22]. In addition, it does not review the literature on LTRAs as an add-on treatment.

In conclusion, tiotropium and LABAs have similar efficacy, and provide greater improvements in lung function than montelukast as add-on to ICS in children and adolescents with asthma. All three controller options have comparable safety profiles. The results of our literature review in patients aged 4–17 years provide needed additional information, and further supports the use of tiotropium in children and adolescents with asthma. The clinical decision on the preferred add-on therapy should also take into account patient phenotype and comorbidities, dose regimen and frequency, the availability of combination therapy, and the delivery device, although more research is required in these younger age groups.

## Data Availability

All data generated or analysed during this study are included in this published article.

## Abbreviations

AE: Adverse event
CI: Confidence interval
FEV_1_: Forced expiratory volume in 1 s
GINA: Global Initiative for Asthma
ICS: Inhaled corticosteroid
LABA: Long-acting β_2_-agonist
LAMA: Long-acting muscarinic antagonist
LTRA: Leukotriene receptor antagonist
OCS: Oral corticosteroid
RCT: Randomised controlled trial
SABA: Short-acting β_2_-agonist
SAE: Serious adverse event
